# Knowledge and Perceptions about Diagnosis, Clinical Management, and Prevention of Dengue Fever among Physicians during the 2023 Outbreak: A Cross-Sectional Study in Peru

**DOI:** 10.4269/ajtmh.23-0794

**Published:** 2024-08-27

**Authors:** Julieta M. Araoz-Salinas, Brando Ortiz-Saavedra, Anderson N. Soriano-Moreno, Martín E. Reategui-Garcia, Carlos Quispe-Vicuña, Valentina Murrieta-Ruiz, Abraham De-Los-Rios-Pinto, Raysa M. Benito-Vargas, Lucero Sangster-Carrasco, Noelia Morocho-Alburqueque, Linda Ponce-Rosas, Jorge Alave, Jose A. Gonzales-Zamora

**Affiliations:** ^1^ Peruvian American Medical Society. Albuquerque, New Mexico;; ^2^ Universidad Nacional Mayor de San Marcos, Lima, Peru;; ^3^ Red de Salud Arequipa—Caylloma, Arequipa, Peru;; ^4^ Clinical and Epidemiological Research Unit, School of Medicine, Universidad Peruana Unión, Lima, Peru;; ^5^ Sociedad Científica de Estudiantes de Medicina de la Amazonía Peruana (SOCIEMAP), Universidad Nacional de la Amazonía Peruana, Iquitos, Peru;; ^6^ Sociedad Científica de San Fernando, Lima, Peru;; ^7^ Escuela Profesional de Medicina Humana, Universidad Nacional de San Antonio Abad del Cusco, Peru;; ^8^ Universidad Peruana de Ciencias Aplicadas, Lima, Peru;; ^9^ Universidad Nacional de Piura, Peru;; ^10^ Department of Medicine, Hamilton Medical Center, Dalton, Georgia;; ^11^ Departamento de Medicina, Facultad de Medicina, Universidad Peruana Unión, Lima, Peru;; ^12^ Division of Infectious Diseases, Department of Medicine, Miller School of Medicine, University of Miami, Miami, Florida

## Abstract

The objective of this study was to evaluate the knowledge level and perception of dengue fever management among Peruvian physicians and to determine the factors associated with higher knowledge. We conducted an analytical cross-sectional study based on an online survey. To evaluate the factors associated with a high level of knowledge (≥70% of correct answers), we used crude (cPR) and adjusted (aPR) prevalence ratios by the Poisson regression model. Of 359 respondents (median age: 33 years; male: 56.5%), 78.8% achieved a high level of knowledge. Multivariable analysis showed an independent association with having read the Peruvian clinical practice guidelines for dengue management (aPR: 1.29; 95% CI: 1.12–1.49), having experience in treating patients (aPR: 1.32; 95% CI: 1.03–1.68), and having treated cases frequently (aPR: 1.22; 95% CI: 1.02–1.46). Residing in the eastern macroregion (aPR: 0.83; 95% CI: 0.71–0.97) was associated with a low level of knowledge. In conclusion, Peruvian physicians had a high level of knowledge about dengue fever. This was associated with having clinical experience in dengue management. However, given the low level of knowledge in the eastern macroregion, educational campaigns are necessary in this area.

## INTRODUCTION

Dengue is a systemic viral infection transmitted by the *Aedes aegypti* and is still a public health problem, especially in tropical and subtropical countries.^1^ Globally, the Americas reported the majority of cases in 2023. There had been more than 3.5 million cases as of August 2023, 1.6 million of which were confirmed, and more than 1,600 deaths in the Americas.^2^ According to the Pan American Health Organization, until August 2023, most cases in the region were reported from Brazil, followed by Peru,^3^ a country that has experienced one of its largest dengue outbreaks in its history, with an increase in the number, severity, and mortality of reported cases and is facing a crisis due to the demand for health services affecting not only adults but also a large number of children.^4^ At the beginning of 2023, 20 of 25 departments in Peru registered confirmed cases of dengue fever, with an increase of 87.3% in the number of cases when compared with the same period in 2022.^5^ In August 2023, Peruvian National Center for Epidemiology, Disease Prevention and Control reported an epidemiological alert given the 398 deaths related to the dengue outbreak (378% higher compared with 2022) during the coastal El Niño phenomenon, which is a recurring climatic pattern characterized by water temperature changes in the north of Peru.^6^^,^^7^

In the fight against dengue’s spread, sociodemographic, environmental, and ecological factors are pivotal.^8^ Nonetheless, it is crucial to emphasize that the first line of defense against the disease hinges on physicians’ knowledge and perception concerning clinical management and infection prevention.^9^ The Peruvian health system is divided into primary care (mostly in rural areas) and secondary and tertiary (both located in urban areas) that attend dengue fever patients according the severity of the disease. For this reason, it is important for the physicians to possess the necessary knowledge to address the ongoing epidemic from different levels of care. Also, early recognition of dengue cases and timely initiation of medical care are crucial because they have been shown to reduce medical complications and mortality in patients with severe dengue.^10^ Likewise, a proper understanding of an infectious agent and its mode of transmission plays a crucial role in the planning and execution of infection control.^11^ Studies evaluating the knowledge level of dengue among physicians in Peru are scarce. A study conducted in Lambayeque region of Peru in 2016 found that 69% of the respondents had a high level of knowledge in physicians.^12^ On the other hand, a study conducted in Ica, southern Peru, which included physicians, odontologists, nurses, and nurse technicians, showed that the level of knowledge about dengue was low.^13^

However, there are no studies assessing the knowledge level of dengue fever in the current 2023 outbreak in Peru. For this reason, it is crucial to conduct studies to provide evidence-based information to bridge gaps and serve as a milestone for policy makers,^14^ aiming to enhance the diagnosis, management, prevention and control for future outbreaks.^15^ The objective of this study was to evaluate the knowledge level and perception of dengue fever regarding the diagnosis, management, and prevention of this disease among Peruvian physicians and to determine the factors associated with higher knowledge. This study will help identify gaps in knowledge and determine the perceptions of the physicians regarding the current public health measures implemented for the 2023 dengue outbreak in Peru. We believe the results will facilitate the development of educational campaigns aimed at reinforcing the necessary concepts for the accurate detection of cases.

## MATERIALS AND METHODS

### Study design, setting, and participants.

We conducted a cross-sectional study from June 21, 2023 to July 20, 2023 in Peru. We adhered to the STROBE checklist for cross-sectional studies (Supplemental Material 1). When the data collection was initiated, there were 187,946 reported cases of dengue in the country, which increased by the end of the study period to 220,990.^16^ In response to the outbreak, the Peruvian Ministry of Health (MINSA) declared a health emergency in the most affected regions of Peru.^17^ Eligible participants included licensed physicians who live and practice Medicine in Peru.

### Sample and diffusion.

Because of the exploratory nature of the study, we determined the sample size based on the total number of licensed physicians in Peru (66,004),^18^ a prevalence of high level of knowledge of 69%, as reported in a previous study,^12^ a 95% CI, and a 5% margin of error. Using these parameters, the sample size was 328 individuals. In addition, we considered 10% of possible surveys that were incorrectly completed; consequently, the minimum number of completed surveys was set at 361. To recruit participants, we used a nonprobabilistic snowball sampling method. We distributed the survey across various social media platforms, including Facebook, Twitter, Instagram, and WhatsApp.

### Questionnaire.

The study used an online survey to gather data from participants (Supplemental Material 2). This survey was created using Google Forms and had the following sections: 1) informed consent, 2) inclusion criteria, 3) sociodemographic data (13 items), 4) clinical experience related to dengue fever (six items), 5) knowledge about clinical management and prevention about dengue fever (13 items), and 6) perceptions about dengue fever (four items). To develop the questionnaire, we used the Peruvian Clinical Practice Guideline for the Clinical Management and Treatment of Dengue approved through Ministerial Resolution No. 071-2017-MINSA^19^ and similar questionnaires on Knowledge, Attitude, and Practice (KAP) studies on dengue used in Peru,^12^ and other countries.^20^^,^^21^ Additionally, we adapted the questions to the Peruvian reality and incorporated specific concepts of the current outbreak in the country. This survey was validated by experts in the field of infectious diseases from Peru and the United States.

### Independent variables.

We evaluated sociodemographic variables such as gender (male, female); age; macro-region of residence (Lima and Callao, Center, North, East, and South); undergraduate university (public, private); years since graduation from medical school; medical position (general practitioner, resident, or specialist); medical specialty; postgraduate degree (doctorate, master’s, or none); area of work (urban, rural), health sector (public, private); workplace (health post, health center, polyclinic, public hospital, private clinic, research center, university). Other variables included knowledge of the current outbreak; having read the Peruvian clinical practice guideline for dengue; training on dengue clinical management in the past 3 months; any medical experience treating dengue, treating dengue cases in the past 3 months; frequency of treating dengue cases. We also evaluated variables related to perceptions toward the Peruvian government measures for the outbreak, and perceptions regarding the dengue fever vaccine.

### Dependent variable.

To define the variable “level of knowledge about clinical management and prevention about dengue fever,” we used 13 items from questions 20 to 32 of the questionnaire, each question had four response alternatives and only one correct answer. If the respondent answered ≥70% of the items correctly, they were considered to have a high level of knowledge. The ≥70% cutoff was based on previous KAP studies.^22^^,^^23^

## STATISTICAL ANALYSES

The statistical analysis was conducted using RStudio version 4. We described participant characteristics using frequencies for categorical variables and mean with standard deviation for numeric variables. In the bivariate analysis, we compared characteristics between the participants who had a high level of knowledge about dengue fever clinical management and prevention and those who did not. We used the Fisher’s exact test and χ^2^ test of independence for categorical variables, and the Wilcoxon rank-sum test for numerical variables. Finally, we calculated crude and adjusted prevalence ratios (PR) with 95% confidence intervals (CIs) using Poisson regression with robust variance. The adjusted model included age group, job location, and all variables with a *P*-value below 0.20 in the crude analysis. Variables with a *P*-value below 0.05 in the adjusted analysis were considered statistically significant associated factors.

## RESULTS

**Figure 1.**
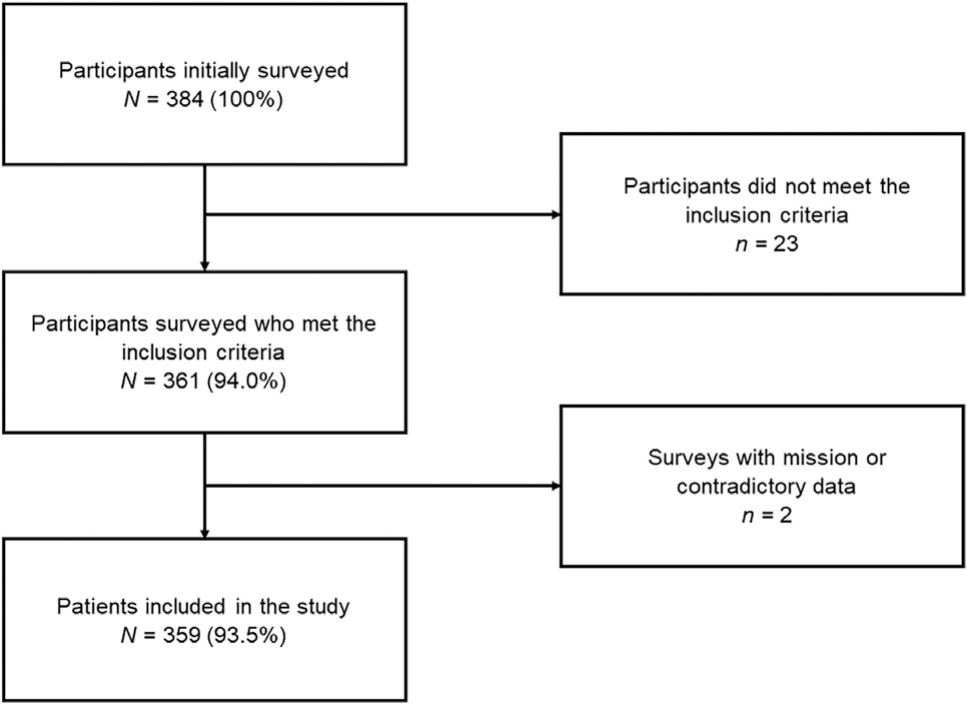
Participant selection flowchart.

Of 384 respondents, 23 did not meet the inclusion criteria, and two submitted incomplete information, resulting in a final sample of 359 (Figure 1). The majority of participants were men (56.5%) between 29 and 49 years of age (49.0%). The majority resided in Lima and Callao (42.9%) and were originally from Peru (98.3%). The majority completed undergraduate studies in Peru (96.4%), at a public university (66.0%), and graduated between 0 and 9 years earlier (67.1%). The majority were general practitioners (71.6%) who worked in the public sector (62.4%) and in urban areas (76.6%). A total of 99.7% were aware of the increase in dengue cases, 65.5% had read the current Peruvian technical guidelines, 52.9% had not received training on dengue management in the past 3 months, 77.7% had seen at least a patient with a confirmed or probable diagnosis of dengue during their entire career, but only 62.4% had seen at least one patient in the past 3 months (Table 1).

**Table 1:** General characteristics of the population

Characteristics	*N* = 359
Sex	
Female	156 (43.5%)
Male	203 (56.5%)
Age, median (SD)	33 (9)
Age	
18–29	157 (43.7%)
30–49	176 (49.0%)
50–75	26 (7.2%)
Macroregion	
Lima and Callao	154 (42.9%)
Center	23 (6.4%)
North	62 (17.3%)
East	69 (19.2%)
South	51 (14.2%)
Country of birth	
Perú	353 (98.3%)
Other countries	6 (1.7%)
Undergraduate studies in Peru	
No	13 (3.6%)
Yes	346 (96.4%)
Type of university where the undergraduate studies were carried out	
Private university	122 (34.0%)
Public university	237 (66.0%)
Years since university graduation	
<1	41 (11.4%)
1–9	241 (67.1%)
>9	77 (21.4%)
Medical position held	
General physician	257 (71.6%)
Resident physician (in training)	37 (10.3%)
Specialist physician	65 (18.1%)
Master’s degree or higher	
No	301 (83.8%)
Yes	58 (16.2%)
Sector in which respondent currently works	
Private	88 (24.5%)
Public	224 (62.4%)
Public and private	47 (13.1%)
Job location	
Rural	84 (23.4%)
Urban	275 (76.6%)
Have you heard about the increase of dengue cases in Peru?	
No	1 (0.3%)
Yes	358 (99.7%)
Have you read the “Peruvian Clinical Practice Guide for the Clinical Management and Treatment of Dengue,” approved by Ministerial Resolution No. 071-2017-MINSA?	
No	124 (34.5%)
Yes	235 (65.5%)
Have you received any training on the clinical management of dengue in Peru in the past 3 months?	
No	190 (52.9%)
Yes	169 (47.1%)
In your medical practice, have you ever treated patients with a confirmed or probable diagnosis of dengue fever?	
No	80 (22.3%)
Yes	279 (77.7%)
Have you treated probable or suspected cases of dengue fever in the past 3 months?	
No	135 (37.6%)
Yes	224 (62.4%)
How often have you treated cases of dengue fever?	
None in the past 3 months	135 (37.6%)
Rarely (1–10 cases/in the past 3 months)	55 (15.3%)
Sometimes (1–10 cases/month)	75 (20.9%)
Frequently (1–10 cases/week or more)	94 (26.2%)
What is your opinion of the following statement? The MINSA is *adequately* training physicians about dengue during the current outbreak.	
Disagree	172 (47.9%)
Neither agree nor disagree	96 (26.7%)
Agree	91 (25.3%)
What is your opinion about the following statement? The MINSA is *adequately *applying vector prevention and control measures (fumigation, elimination of mosquito breeding sites, etc.)	
Disagree	182 (50.7%)
Neither agree nor disagree	100 (27.9%)
Agree	77 (21.4%)
What do you think of the following statement? The MINSA should implement vaccination against dengue in the country.	
Disagree	35 (9.7%)
Neither agree nor disagree	68 (18.9%)
Agree	256 (71.3%)
What is your opinion of the following statement? The MINSA has the necessary resources to deal with dengue cases in the current outbreak.	
Disagree	227 (63.2%)
Neither agree nor disagree	67 (18.7%)
Agree	65 (18.1%)
Percentage of correct answers	
<70%	76 (21.2%)
≥70%	283 (78.8%)

MINSA, Peruvian Ministry of Health.

Data are *n* (%) unless otherwise noted.

Regarding knowledge, 78.8% obtained more than 70% correct answers. Almost all participants knew about *Aedes aegypti* as a transmitter (99.7%), incubation period (81.6%), stages of the disease (87.7%), recognized a probable case of dengue (98.6%), knew about paracetamol for symptomatic treatment (97.5%), and knew that there was currently no vaccine against dengue in Peru (93.9%). Also, only 59.6% of the participants were aware of the U.S. Food and Drug Administration (FDA)-approved vaccine (Table 2).

**Table 2:** Percentage of correct answers for each question of the questionnaire

Question	*N* = 359
Q20. How is dengue transmitted?	358 (99.7%)
Q21. What time of day are people most likely to become infected with dengue?	177 (49.3%)
Q22. What is the incubation period of dengue?	293 (81.6%)
Q23. After the incubation period, the disease is followed by the next three phases:	315 (87.7%)
Q24. Which of the following cases can be classified as a probable case of dengue fever?	354 (98.6%)
Q25. A 25-year-old female patient presented 3 days ago with fever, retroocular headache, persistent nausea and vomiting. Physical examination revealed a generalized skin rash. Hemoglobin level was 17 g/dL, hematocrit 52%, leukocytes 2,000/mm^3^, and platelets 110,000 uL/mm^3^. In this clinical case, what are the warning signs that you identify?	265 (73.8%)
Q26. What clinical manifestation is indicative of severe dengue?	265 (73.8%)
Q27. When is it recommended to perform the NS1 antigen detection test for the diagnosis of dengue?	307 (85.5%)
Q28. When is it most likely to obtain a positive IgM serological test result in a case of dengue?	287 (79.9%)
Q29. What symptomatic treatment would you give to a patient with dengue without warning signs?	350 (97.5%)
Q30. What treatment should be initiated to treat shock in severe dengue?	320 (89.1%)
Q31. Is there a Food and Drug Administration (FDA)-approved vaccine in the world to prevent dengue?	214 (59.6%)
Q32. Is a vaccine against dengue currently available in Peru?	337 (93.9%)

**Figure 2.**
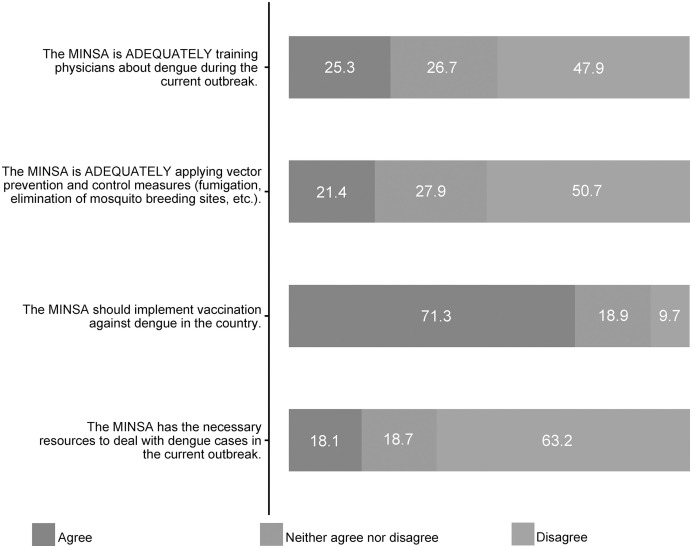
Perceptions of the physicians regarding the current public health measures implemented for the dengue outbreak in Peru. MINSA, Peruvian Ministry of Health.

Regarding perception, 50% considered that MINSA was not adequately training physicians during the current outbreak (47.9%), that it was not adequately applying vector prevention and control measures (50.7%), and that it did not have the necessary resources (63.2%). Likewise, most of the participants believed that MINSA should have implemented vaccination against dengue (71.3%) (Figure 2).

A score ≥70% was significantly higher among northern residents (*P* = 0.021), who had previously read the Peruvian guideline (*P* <0.001), who received training in the clinical management of dengue in the past 3 months (*P* = 0. 001), who had ever treated patients with a confirmed or probable diagnosis of dengue (*P* <0.001), who had treated cases in the past 3 months (*P* <0.001; rarely, *P* = 0.004; sometimes, 0.001; and frequently, *P* <0.001). Regarding perceptions, a score ≥70% was significantly higher in those who believed that MINSA was adequately training dengue physicians during the current outbreak (*P* = 0.008) and that it should have implemented dengue vaccination (*P* = 0.037) (Table 3).

**Table 3:** Characteristics of the population according to percentage of knowledge (≥70%).

Characteristics	<70%	≥70%	cPR (95% CI)	*P*-Value
	*N* = 76	*N* = 283		
Sex				
Female	34 (21.8%)	122 (78.2%)	Ref	Ref
Male	42 (20.7%)	161 (79.3%)	1.01 (0.91–1.13)	0.799
Age, median (SD)	33.9 (10.2)	33.1 (8.37)	1.00 (0.99–1.00)	0.597
Age				
18–29	31 (19.7%)	126 (80.3%)	Ref	Ref
30–49	39 (22.2%)	137 (77.8%)	0.97 (0.87–1.08)	0.594
50–75	6 (23.1%)	20 (76.9%)	0.96 (0.77–1.20)	0.684
Macroregion				
Lima and Callao	32 (20.8%)	122 (79.2%)	Ref	Ref
Center	4 (17.4%)	19 (82.6%)	1.04 (0.85–1.28)	0.743
North	5 (8.06%)	57 (91.9%)	1.16 (1.04–1.29)	0.021
East	15 (21.7%)	54 (78.3%)	0.99 (0.85–1.15)	0.863
South	20 (39.2%)	31 (60.8%)	0.77 (0.61–0.97)	0.012
Country of birth				
Perú	75 (21.2%)	278 (78.8%)	Ref	Ref
Other countries	1 (16.7%)	5 (83.3%)	1.06 (0.74–1.52)	0.864
Undergraduate studies in Peru				
No	4 (30.8%)	9 (69.2%)	Ref	Ref
Yes	72 (20.8%)	274 (79.2%)	1.14 (0.79–1.65)	0.404
Type of university where undergraduate studies were carried out				
Private university	21 (17.2%)	101 (82.8%)	Ref	Ref
Public university	55 (23.2%)	182 (76.8%)	0.93 (0.83–1.03)	0.190
Years since university graduation				
<1	13 (31.7%)	28 (68.3%)	Ref	Ref
1–9	45 (18.7%)	196 (81.3%)	1.19 (0.96–1.48)	0.069
>9	18 (23.4%)	59 (76.6%)	1.12 (0.88–1.43)	0.339
Medical position held				
General physician	47 (18.3%)	210 (81.7%)	Ref	Ref
Resident physician (in training)	12 (32.4%)	25 (67.6%)	0.83 (0.66–1.04)	0.058
Specialist physician	17 (26.2%)	48 (73.8%)	0.90 (0.77–1.06)	0.167
Master’s degree or higher				
No	63 (20.9%)	238 (79.1%)	Ref	Ref
Yes	13 (22.4%)	45 (77.6%)	0.98 (0.84–1.14)	0.787
Sector in which they currently work				
Private	14 (15.9%)	74 (84.1%)	Ref	Ref
Public	54 (24.1%)	170 (75.9%)	0.90 (0.80–1.01)	0.114
Public and private	8 (17.0%)	39 (83.0%)	0.99 (0.84–1.16)	0.860
Job location				
Rural	22 (26.2%)	62 (73.8%)	Ref	Ref
Urban	54 (19.6%)	221 (80.4%)	1.09 (0.95–1.25)	0.206
Have you heard about the increase of dengue cases in Peru?				
No	0 (0.00%)	1 (100%)	Ref	Ref
Yes	76 (21.2%)	282 (78.8%)	0.79 (0.75–0.83)	0.788
Have you read the “Peruvian Clinical Practice Guide for the Clinical Management and Treatment of Dengue,” approved by Ministerial Resolution No. 071-2017-MINSA?				
No	48 (38.7%)	76 (61.3%)	Ref	Ref
Yes	28 (11.9%)	207 (88.1%)	1.44 (1.24–1.67)	<0.001
Have you received any training on the clinical management of dengue in Peru in the last 3 months?				
No	53 (27.9%)	137 (72.1%)	Ref	Ref
Yes	23 (13.6%)	146 (86.4%)	1.20 (1.08–1.33)	0.001
In your medical practice, have you ever treated patients with a confirmed or probable diagnosis of dengue fever?				
No	36 (45.0%)	44 (55.0%)	Ref	Ref
Yes	40 (14.3%)	239 (85.7%)	1.56 (1.27–1.91)	<0.001
Have you treated probable or suspected cases of dengue fever in the past 3 months?				
No	50 (37.0%)	85 (63.0%)	Ref	Ref
Yes	26 (11.6%)	198 (88.4%)	1.40 (1.22–1.61)	<0.001
How often have you treated cases of dengue fever?				
Not treated in the past 3 months	50 (37.0%)	85 (63.0%)	Ref	Ref
Rarely (1–10 cases/in the past 3 months)	9 (16.4%)	46 (83.6%)	1.33 (1.12–1.58)	0.004
Sometimes (1–10 cases/month)	12 (16.0%)	63 (84.0%)	1.33 (1.13–1.57)	0.001
Frequently (1–10 cases/week or more)	5 (5.32%)	89 (94.7%)	1.50 (1.31–1.73)	<0.001
What is your opinion of the following statement? The Peruvian Ministry of Health is *adequately* training physicians about dengue during the current outbreak.				
Neither agree nor disagree	28 (29.2%)	68 (70.8%)	Ref	Ref
Disagree	36 (20.9%)	136 (79.1%)	1.12 (0.96–1.30)	0.136
Agree	12 (13.2%)	79 (86.8%)	1.23 (1.05–1.43)	0.008
What is your opinion about the following statement? The MINSA is *adequately* applying vector prevention and control measures (fumigation, elimination of mosquito breeding sites, etc.)				
Neither agree nor disagree	25 (25.0%)	75 (75.0%)	Ref	Ref
Disagree	37 (20.3%)	145 (79.7%)	1.06 (0.93–1.22)	0.370
Agree	14 (18.2%)	63 (81.8%)	1.09 (0.93–1.27)	0.286
What do you think of the following statement? The MINSA should implement vaccination against dengue in the country.				
Neither agree nor disagree	20 (29.4%)	48 (70.6%)	Ref	Ref
Disagree	11 (31.4%)	24 (68.6%)	0.97 (0.74–1.27)	0.830
Agree	45 (17.6%)	211 (82.4%)	1.17 (0.99–1.38)	0.037
What is your opinion of the following statement? The MINSA has the necessary resources to deal with dengue cases in the current outbreak.				
Neither agree nor disagree	19 (28.4%)	48 (71.6%)	Ref	Ref
Disagree	47 (20.7%)	180 (79.3%)	1.11 (0.94–1.30)	0.196
Agree	10 (15.4%)	55 (84.6%)	1.18 (0.98–1.42)	0.077

cPR = crude prevalence ratio; MINSA: Peruvian Ministry of Health; Ref = reference.

In the adjusted multivariate model, it was observed that having read the MINSA clinical practice guidelines (adjusted prevalence ratio [aPR]: 1.29; 95% CI: 1.12–1.49), having treated patients diagnosed with dengue (aPR: 1.32; 95% CI: 1.03–1.68), having frequently treated dengue cases (aPR: 1.22; 95% CI: 1.02–1.46), and believing that MINSA adequately training medical personnel on dengue (agree aPR: 1.23; 95% CI: 1.05–1.44, or disagree aPR: 1.20; 95% CI: 1.04–1.38) were independently associated with high knowledge (≥70%). On the other hand, residing in the eastern macroregion (aPR: 0.83; 95% CI: 0.71–0.97) was associated with low knowledge (<70%) (Table 4).

**Table 4:** Multivariate analysis of factors associated with a percentage of knowledge ≥70

Factors	Knowledge ≥70%		
	aPR	95% CI	*P*-Value
Age			
18–29	1.00		
30–49	1.00	0.89–1.13	0.978
50–75	1.02	0.80–1.31	0.843
Macroregion			
Lima and Callao	1.00		
Center	0.95	0.77–1.16	0.605
North	0.96	0.84–1.09	0.499
East	0.83	0.71–0.97	**0.017**
South	0.95	0.76–1.19	0.651
Type of university where the undergraduate studies were carried out			
Private university	1.00		
Public university	1.04	0.93–1.17	0.451
Years since university graduation			
<1	1.00		
1–9	1.00	0.81–1.23	0.983
>9	0.96	0.73–1.27	0.788
Medical position held			
General physician	1.00		
Resident physician (in training)	0.84	0.69–1.03	0.103
Specialist physician	0.84	0.70–1.01	0.066
Sector in which they currently work			
Private	1.00		
Public	0.88	0.77–1.00	0.057
Public and private	0.98	0.83–1.15	0.780
Job location			
Rural	1.00		
Urban	0.99	0.86–1.14	0.883
Have you read the “Peruvian Clinical Practice Guide for the Clinical Management and Treatment of Dengue,” approved by Ministerial Resolution No. 071-2017-MINSA?			
No	1.00		
Yes	1.29	1.12–1.49	**0.001**
Have you received any training on the clinical management of dengue in Peru in the past 3 months?			
No	1.00		
Yes	1.00	0.89–1.13	0.943
In your medical practice, have you ever treated patients with a confirmed or probable diagnosis of dengue fever?			
No	1.00		
Yes	1.32	1.03–1.68	**0.027**
How often have you treated cases of dengue fever?			
No cases in the past 3 months	1.00		
Rarely (1–10 cases/in the past 3 months)	1.08	0.89–1.31	0.425
Sometimes (1–10 cases/month)	1.11	0.92–1.33	0.297
Frequently (1–10 cases/week or more)	1.22	1.02–1.46	**0.030**
What is your opinion of the following statement? The MINSA is *adequately* training physicians about dengue during the current outbreak.			
Disagree	1.20	1.04–1.38	**0.015**
Neither agree nor disagree	1.00		
Agree	1.23	1.05–1.44	**0.009**
What do you think of the following statement? The MINSA should implement vaccination against dengue in the country.			
Disagree	0.98	0.76–1.26	0.876
Neither agree nor disagree	1.00		
Agree	1.13	0.97–1.31	0.119
What is your opinion of the following statement? The MINSA has the necessary resources to deal with dengue cases in the current outbreak.			
Disagree	1.04	0.89–1.21	0.639
Neither agree nor disagree	1.00		
Agree	1.10	0.92–1.31	0.320

aPR = adjusted prevalence ratio; MINSA = Peruvian Ministry of Health.

*P*-values in bold are statistically significant (*P*-value < 0.05).

## DISCUSSION

This is the first exploratory study that evaluated the level of knowledge regarding the diagnosis, management, and prevention of dengue among physicians from different regions of Peru during the 2023 outbreak. The majority of participants (78.8%) had at least 70% correct answers. In a national context, a 2016 study conducted in the province of Lambayeque, Peru, found that 69% of doctors obtained an average of nine to 16 correct answers, which was considered to be a good level of knowledge.^12^ Dengue is a well-known disease among doctors in Peru because it is an endemic disease in several provinces, such as Lambayeque.^12^^,^^24^ In an international context, some studies have revealed a high level of knowledge related to dengue. For example, a pre-symposium survey in Florida^25^ showed physicians scoring an average of 74.3% on dengue-related questions. In Tanzania, Saringe et al.^26^ found that 74.1% of healthcare workers possessed good dengue knowledge, with a mean score of 43.6 (considered good if ≥40). Additionally, a multicountry study revealed that Bangladesh, India, and Malaysia had the highest dengue knowledge scores (16.9, 18.03, and 18.46, respectively), whereas Turkey scored lower (11.7).^27^ These studies suggest that the good knowledge could be due to the constant dengue cases presented in those areas, a situation similar to Peru.

In the multivariate analysis, it was found that reading the current Peruvian clinical practice guidelines for dengue management was positively associated with obtaining a score ≥70%. These results are in agreement with those reported by Handel et al.,^28^ who assessed KAP about dengue infection in health personnel in Machala, Ecuador, and found a higher score in workers who were more familiar with the 2009 WHO dengue guidelines. In both studies, clinical practice guidelines were used as a reference to perform the respective knowledge questionnaire. Furthermore, the importance of knowledge of clinical practice guidelines, whether international or local, lies in the fact that they reduce unjustified variation in practice and improve the quality of medical care.^29^ This premise is reinforced by the fact that in 2022 in Peru, according to an analysis of medical records of dengue deaths, there was evidence of a lack of adherence to the 2017 Clinical Practice Guideline for the care of dengue in Peru by the health personnel who attended the patients.^30^ Therefore, it is important to know the local clinical practice guidelines to avoid unfavorable outcomes in the care of patients with dengue.

Our study also found that physicians who treated at least one dengue case in the previous 3 months had a higher prevalence of knowledge ≥70% compared with those who did not. Ruberto et al.^31^ found similar results. In their study, the doctors who had heard about the increasing number of dengue cases in Sonora, Mexico, had higher prevalence of good knowledge compared with those who did not. These results may suggest that doctors who frequently treat dengue cases or who learned about the increase in the number of cases were more likely to have had to research and update their knowledge about the pathophysiology, diagnosis, and treatment to manage cases or prepare for the possible arrival of cases at their health centers.

Differences in dengue knowledge scores by geographic location have been previously compared in physicians from endemic and nonendemic countries.^20^^,^^27^^,^^32^ However, this study divided respondents according to macroregions within the same country, and despite the fact that the Eastern macroregion tends to have the highest number of cases and is considered a dengue endemic area,^33^ in the multivariable analysis, living in this macroregion was negatively associated with obtaining a score ≥70%. Similarly, physicians in dengue-endemic countries such as Taiwan and Puerto Rico were found to have lower scores on their dengue knowledge surveys.^32^^,^^34^ This is contradictory because physicians from endemic areas, who treat more dengue patients in their daily clinical practice, are expected to have higher scores on dengue knowledge tests.^25^ It could be explained that in endemic areas such as Peru’s eastern region, doctors may often rely more on empirical knowledge from their daily experiences rather than adhering to the current national technical guidelines and provided trainings. A comprehensive study is needed to understand the factors contributing to this situation. Nevertheless, the results of this study are inconclusive because doctors from the eastern macroregion accounted for only 19.2% of the sample. We believe this variation should be studied further with a larger sample.

We also found that physicians who clearly expressed their viewpoint on whether the MINSA provided adequate training for healthcare personnel, whether in agreement or disagreement, showed a higher level of knowledge regarding dengue compared with those who remained neutral on the issue. Physicians who agreed probably believed they had received adequate training by MINSA on dengue and therefore might have felt more confident diagnosing and treating dengue. This premise is consistent with the results of a study conducted by the Florida Department of Health, where a significant increase in self-confidence in recognizing dengue cases was reported, with assessment scores increasing from a pretest mean score of 4.1 to a posttest mean score of 7 out of 10.^25^ In cases of disagreement, participants may have received different training sessions from MINSA, enabling them to compare and provide an opinion on this matter; however, there are no studies for evaluating dengue clinical management programs offered by MINSA. In an international context, the Secretary of Health of Puerto Rico presented a training course on dengue management from the Dengue Branch of the CDC and evaluated physicians’ perceptions of the impact of the course on medical practice in their hospitals. All respondents noted positive changes and appreciated the content and importance of the course.^35^ Therefore, conducting studies to evaluate the training programs offered by healthcare institutions is crucial for enhancing the practices in managing dengue fever and preventing deaths.^36^

Regarding knowledge gaps in our population, it was noticed that less than a half of the participants (49%) correctly identified the feeding time behavior of *Aedes aegypti* as during the early morning and early evening. Divergent results were found across different studies in the literature. Higher rates of correct answers were reported in Ethiopia, Iran, and Ecuador, with percentages of 79%,^37^ 75.7%,^38^ and 75%,^28^ respectively. In contrast, lower percentages were found in a study in Taiwan 27.1%.^39^ Although in our study, the percentage falls within the midrange of correct responses compared with other studies, it underscores the need for continued efforts to enhance this concept. Having a good knowledge of *Aedes* biting behavior is important for physicians given its implications for preventive measures.

Also, we found that almost 60% of the physicians were aware of the existence of the dengue vaccine. Similar results were found in Bhutan^40^ where 58.8% of the participants were aware of the dengue vaccine and its indication for the population aged ≥9 years. Additionally, 71.3% of the participants felt that the MINSA should implement vaccination against dengue in the country. Despite these results, there are factors that may reduce the vaccine applicability in Peruvian medical practice, such as the need for greater resources,^41^ the requirement for laboratory testing, and limitations in conducting adequate trials.^42^ However, there are some countries in Latin America that already approved the dengue vaccine, such as Brazil and Argentina, where the Qdenga vaccine has been administered since 2023.^43^^,^^44^ On the other hand, the Peruvian government is still evaluating whether to acquire the vaccine against dengue in 2024.^45^ We believe it is crucial to use of dengue vaccine in endemic countries to mitigate the impact of future outbreaks.

### Perception related to management of dengue fever in Peru.

In the perception domain, 47.9% of the participants disagreed with the premise that the MINSA is adequately training physicians on dengue during the current outbreak. We think that the poor management of the COVID-19 pandemic in Peru could have undermined the credibility of the Peruvian government among physicians, particularly after the approximately 400 physicians death^46^ and the “Vacuna-gate” scandal^47^ that occurred during early 2021. This was a case of corruption that involved the irregular inoculation of the Sinopharm vaccine against COVID-19 during its experimental stage, benefiting the elite instead of prioritizing the population at higher risk. Other important findings were that 50.7% of participants disagreed with the premise that MINSA is adequately implementing prevention and vector control measures, and 63.2% of participants disagreed with the premise that MINSA has the necessary resources to address dengue cases in the current outbreak. It could be attributable to the unstable government, which has become a feature of Peruvian politics with the country having six health ministers in the past 2 years.^48^ This instability has made it difficult to establish political health policies according to the population’s necessities. Further, the conjunction of rainy conditions, substantial precipitation, and flooding in Peru, intensified by the presence of cyclone Yaku (the first cyclone recorded in the country in four decades) in March 2023, provided an ideal setting for the recent dengue outbreak in the nation.^4^

### Limitations and strengths.

There are some limitations to our study. Our sample was not fully representative of the entire population of physicians in Peru. Because the study was based on an online survey, only physicians with Internet access were able to participate. Additionally, most physicians who work in rural areas could not answer this survey, resulting in a selection bias. For this reason, we suggest that future studies include healthcare professionals from rural areas to gather a more diverse sample. Furthermore, our study had a majority of male participants aged 29 to 49, indicating gender bias. To improve representativeness in future studies, we recommend including participants across diverse ages and regions in Peru. Also, nonprobability sampling was used to enroll participants, making it difficult to extrapolate the results. Another limitation was the possibility of dishonesty in answering the questions, a factor that could not be completely excluded because of the lack of supervision of the participants during the survey. Despite these limitations, the present study is the first to assess physician knowledge and perception of dengue in the current outbreak in Peru. In addition, our study involved a sample of all macroregions of Peru to gain a better understanding of the differences between each macro region.

## CONCLUSION

The level of knowledge about dengue fever was high among physicians in Peru, but gaps exist, such as timing of infection risk and the existence of an FDA-approved vaccine. On the other hand, physicians who lived in the eastern macroregion had lower knowledge, suggesting that educational campaigns might be needed in eastern Peru to improve the detection and management of cases. Our study also showed that having clinical experience in dengue is associated with a higher level of knowledge. We believe that physicians with clinical expertise in dengue should lead the educational programs for healthcare workers in Peru and be considered among the key actors in the development of public health policies.

## Supplemental Materials

10.4269/ajtmh.23-0794 Supplemental Materials
