# Supplementary material for: Knowledge and Perceptions about Diagnosis, Clinical Management, and Prevention of Dengue Fever among Physicians during the 2023 Outbreak: A Cross-Sectional Study in Peru
Source: Am J Trop Med Hyg. 2024 Aug 27;111(5):1082–92. doi: 10.4269/ajtmh.23-0794 (PMC11542531; doi:10.4269/ajtmh.23-0794)
Supplement: Supplemental Materials [file tpmd230794.SD1.pdf]

Supplementary material 1: STROBE Statement—Checklist of items that should be included in reports of cross-sectional studies.

	Item No	Recommendation
**Title and abstract**	1	(*a*) Indicate the study's design with a commonly used term in the title or the abstract **(p. 01)**
		(*b*) Provide in the abstract an informative and balanced summary of what was done and what was found **(p. 04)**
**Introduction**		
Background/rationale	2	Explain the scientific background and rationale for the investigation being reported **(p. 05-06)**
Objectives	3	State specific objectives, including any prespecified hypotheses **(p. 07)**
**Methods**		
Study design	4	Present key elements of study design early in the paper **(p. 07)**
Setting	5	Describe the setting, locations, and relevant dates, including periods of recruitment, exposure, follow-up, and data collection **(p. 07-08)**
Participants	6	(*a*) Give the eligibility criteria, and the sources and methods of selection of participants **(p. 07)**
Variables	7	Clearly define all outcomes, exposures, predictors, potential confounders, and effect modifiers. Give diagnostic criteria, if applicable **(p. 08-09)**
Data sources/ measurement	8*	For each variable of interest, give sources of data and details of methods of assessment (measurement). Describe comparability of assessment methods if there is more than one group **(p. 08-09)**
Bias	9	Describe any efforts to address potential sources of bias
Study size	10	Explain how the study size was arrived at **(p. 07)**
Quantitative variables	11	Explain how quantitative variables were handled in the analyses. If applicable, describe which groupings were chosen and why
Statistical methods	12	(*a*) Describe all statistical methods, including those used to control for confounding **(p. 09)**
		(*b*) Describe any methods used to examine subgroups and interactions
		(*c*) Explain how missing data were addressed
		(*d*) If applicable, describe analytical methods taking account of sampling strategy
		(*e*) Describe any sensitivity analyses
**Results**		
Participants	13*	(a) Report numbers of individuals at each stage of study—eg numbers potentially eligible, examined for eligibility, confirmed eligible, included in the study, completing follow-up, and analysed **(p. 10)**
		(b) Give reasons for non-participation at each stage **(p. 10, 27)**
		(c) Consider use of a flow diagram **(p. 27)**
Descriptive data	14*	(a) Give characteristics of study participants (eg demographic, clinical, social) and information on exposures and potential confounders **(p. 10-11)**
		(b) Indicate number of participants with missing data for each variable of interest
Outcome data	15*	Report numbers of outcome events or summary measures **(p. 10-11)**

Main results	16	(*a*) Give unadjusted estimates and, if applicable, confounder-adjusted estimates and their precision (eg, 95% confidence interval). Make clear which confounders were adjusted for and why they were included **(p. 11-12)**
		(*b*) Report category boundaries when continuous variables were categorized
		(*c*) If relevant, consider translating estimates of relative risk into absolute risk for a meaningful time period
Other analyses	17	Report other analyses done—eg analyses of subgroups and interactions, and sensitivity analyses
**Discussion**		
Key results	18	Summarise key results with reference to study objectives
Limitations	19	Discuss limitations of the study, taking into account sources of potential bias or imprecision. Discuss both direction and magnitude of any potential bias **(p. 17)**
Interpretation	20	Give a cautious overall interpretation of results considering objectives, limitations, multiplicity of analyses, results from similar studies, and other relevant evidence **(p. 12:16)**
Generalisability	21	Discuss the generalisability (external validity) of the study results **(p. 17)**
**Other information**		
Funding	22	Give the source of funding and the role of the funders for the present study and, if applicable, for the original study on which the present article is based **(p. 18)**

*Give information separately for exposed and unexposed groups.

Supplementary material 2. Survey to assess Knowledge and Perceptions about the Diagnosis, Clinical Management, and Prevention of Dengue Fever

Inclusion Criteria

1. **Are you a licensed doctor in Peru?**

    a. Yes

    b. No (survey ends)

2. **Do you currently live in Peru?**

    a. Yes

    b. No (survey ends)

3. **Are you currently practicing human medicine in Peru (including clinical, research, administrative, and teaching roles)**

    a. Yes

    b. No (survey ends)

Basic participant information

1. **Please indicate your gender:**

    a. Male

    b. Female

2. **What is your age (in years and numbers)? ______**

3. **What is your country of birth?**

    a. Perú

    b. Venezuela

    c. Bolivia

    d. Argentina

    e. Ecuador

    f. Colombia

    g. Chile

    h. Brasil

    i. Paraguay

j. Otro, especifique: ___________

4. Please indicate the department where you currently reside:

a. Lima

b. Callao

c. Arequipa

d. Loreto

e. Ica

f. La Libertad

g. Ancash

h. Junín

i. Cajamarca

j. Apurímac

k. Ayacucho

l. Cusco

m. Huancavelica

n. Huánuco

o. Lambayeque

p. Madre de Dios

q. Moquegua

r. Pasco

s. Piura

t. Puno

u. San Martin

v. Tacna

w. Tumbes

x. Ucayali

y. Amazonas

5. Did you complete your undergraduate medical studies in Peru?

a. Yes

b. No

6. What type of university did you attend for your undergraduate studies?

a. Public university

b. Private university

7. How many years have you graduated from the Human Medicine undergraduate program (in years and numbers)? ________

8. Please indicate the medical position you hold:

a. General physician

b. Resident physician (In training)

c. Specialist physician

9. What is your medical specialization?

a. I do not have a medical specialization

b. Internal Medicine

c. Emergency and Disaster Medicine

d. Family Medicine

e. Infectious and Tropical Diseases

f. Intensive Care

g. Pulmonology

h. General Surgery or another surgical specialty

i. Gynecology

j. Pediatrics

k. Other specialization, Specify..........

10. Please indicate if you hold any of the following academic degrees:

a. Master's degree

b. PhD

c. Both (a and b)

d. None

11. In which sector do you currently work? (You can select more than one option)

a. Ministry of Health (MINSA)

b. EsSalud

c. Armed Forces or Police

d. Private

12. Please indicate your current workplace (you can select more than one option)a. Health post

b. Health center

c. Polyclinic

d. Public hospital

e. Private clinic

f. Research center

g. University

h. Other, specify: __________

13. In which region is your workplace located

a. Urban

b. Rural

14. Have you heard about the increase in dengue cases in Peru?

a. Yes

b. No

15. Have you READ the "Peruvian Clinical Practice Guide for the Clinical Management and Treatment of Dengue approved through Ministerial Resolution No. 071-2017-MINSA"?

a. Yes

b. No

16. Have you received any training on the clinical management of dengue in Peru in the last three months?

a. Yes

b. No

17. Have you ever, in the practice of the medical profession, treated patients with confirmed or probable dengue diagnosis?

a. Yes

b. No

18. Have you treated probable or suspected dengue cases in the last 3 months?

a. Yes (Proceed to question 19)

b. No

19. How often have you treated dengue cases?

a. Very frequently (more than 10 cases/week)

b. Frequently (1-10 cases/week)

c. Sometimes (1-10 cases/month)

d. Rarely (1-10 cases/in the last three months)

KNOWLEDGE: Please mark the answer you believe is correct

20. How is dengue transmitted?

a. Bite of the Aedes aegypti mosquito (CORRECT)

b. Bite of the Anopheles mosquito

c. Direct contact with an infected person

d. Ingestion of contaminated food

21. At what time of day are people most likely to get infected with dengue?

a. Early morning and early evening (CORRECT)

b. Late afternoon and late morning

c. Early afternoon and late evening

d. Late morning and early evening

22. How long is the incubation period of dengue?

a. 1 to 3 days

b. 3 to 14 days (CORRECT)

c. 15 to 20 days

d. More than 30 days

23. After the incubation period, the disease is followed by the following three phases:

a. Febrile, critical, convalescent (CORRECT)

b. Prodromal, critical, convalescent

c. Febrile, convalescent, stationary

d. Prodromal, Febrile, recovery

24. Which of the following cases can be classified as a probable case of dengue?

a. Patient with fever, dysuria, and lumbar pain.

b. Patient from an endemic area with 2 to 7 days of fever associated with headache and retroocular pain. (CORRECT)

c. Patient with fever, weakness, and marked swelling in the knee.

d. Patient with fever, cough, nasal congestion, and sore throat.

25. A 25-year-old female patient has had fever, retro-ocular headache, nausea, and persistent vomiting for 3 days. Physical examination reveals a generalized rash. The hemogram shows hemoglobin of 17 g/dL, Hematocrit of 52%, Leukocytes at 2000/mm3, and platelets at 110,000 uL/mm3. In this clinical case, what are the warning signs that you identify?

a. Generalized rash and leukopenia

b. Retro-ocular headache and thrombocytopenia

c. Fever for 3 days and thrombocytopenia

d. Persistent vomiting and elevated hematocrit (CORRECT)

26. Which clinical manifestation is indicative of severe dengue?

a. Hematemesis (CORRECT)

b. Petechiae

c. Polyuria

d. Paralysis of lower limbs

27. When is it recommended to perform the NS1 antigen detection test for dengue diagnosis?

a. After 7 days of symptom onset.

b. Immediately after the mosquito bite.

c. After 2 weeks of symptom onset.

d. Within the first 7 days of symptom onset. (CORRECT)

28. When is it most likely to get a positive result in the IgM serological test in a dengue case?

a. After the fifth day of symptoms (CORRECT)

b. The day after mosquito bite

c. In the first 3 days of illness

d. At any time during the course of the disease

29. What SYMPTOMATIC TREATMENT would you give to a dengue patient without warning signs?

a. Paracetamol (CORRECT)

b. Ibuprofen

c. Aspirin

d. Ivermectin

30. What treatment should be initiated to treat shock in severe dengue cases?

a. 5% Dextrose

b. 0.9% Sodium Chloride (CORRECT)

c. Packed red blood cell transfusion

d. Platelet transfusion

31. Is there a vaccine approved by the Food and Drug Administration (FDA) worldwide to prevent dengue?

a. Yes (CORRECT)

b. No

32. Is there currently a dengue vaccine available in Peru?

a. Yes

b. No (CORRECT)

PERCEPTIONS:

33. What is your opinion on the following statement? The Ministry of Health of Peru is ADEQUATELY training doctors about dengue during the current outbreak.

a. I totally agree

b. I agree

c. Neither agree nor disagree

d. I disagree

e. I totally disagree

34. What is your opinion on the following statement? The Ministry of Health of Peru is ADEQUATELY applying vector prevention and control measures (fumigation, mosquito breeding site elimination, etc.).

a. I totally agree

b. I agree

c. Neither agree nor disagree

d. I disagree

e. I totally disagree

35. What is your opinion on the following statement? The Ministry of Health of Peru should implement dengue vaccination in the country.

a. I totally agree

b. I agree

c. Neither agree nor disagree

d. I disagree

e. I totally disagree

36. What is your opinion on the following statement? The Ministry of Health of Peru has the necessary resources to manage dengue cases in the current outbreak.

a. I totally agree

b. I agree

c. Neither agree nor disagree

d. I disagree

e. I totally disagree

Supplementary material 3. Percentage of correct answers for each question of the questionnaire.

Question	N = 359
Q20. How is dengue transmitted?	358 (99.7%)
Q21. What time of day are people most likely to become infected with dengue?	177 (49.3%)
Q22. What is the incubation period of dengue?	293 (81.6%)
Q23. After the incubation period, the disease is followed by the next three phases:	315 (87.7%)
Q24. Which of the following cases can be classified as a probable case of dengue fever?	354 (98.6%)
Q25. A 25-year-old female patient presented 3 days ago with fever, retroocular headache, persistent nausea and vomiting. Physical examination revealed a generalized skin rash. Hemoglobin level was 17 g/dL, hematocrit 52%, leukocytes 2000/mm3 and platelets 110 000 uL/mm3. In this clinical case, what are the warning signs that you identify?	265 (73.8%)
Q26. What clinical manifestation is indicative of severe dengue?	265 (73.8%)
Q27. When is it recommended to perform the NS1 antigen detection test for the diagnosis of dengue?	307 (85.5%)
Q28. When is it most likely to obtain a positive IgM serological test result in a case of dengue?	287 (79.9%)
Q29. What SYMPTOMATIC TREATMENT would you give to a patient with dengue without warning signs?	350 (97.5%)
Q30. What treatment should be initiated to treat shock in severe dengue?	320 (89.1%)
Q31. Is there a Food and Drug Administration (FDA) approved vaccine in the world to prevent dengue?	214 (59.6%)
Q32. Is a vaccine against dengue currently available in Peru?	337 (93.9%)

Supplementary material 4. Characteristics of the population according to percentage of knowledge (≥70%).

	<70% N=76	≥70% N=283	cPR (95% CI)	p-value
Gender				
Female	34 (21.8%)	122 (78.2%)	Ref.	Ref.
Male	42 (20.7%)	161 (79.3%)	1.01 (0.91-1.13)	0.799
Age[1]	33.9 (10.2)	33.1 (8.37)	1.00 (0.99-1.00)	0.597
Age				
18-29	31 (19.7%)	126 (80.3%)	Ref.	Ref.
30-49	39 (22.2%)	137 (77.8%)	0.97 (0.87-1.08)	0.594
50-75	6 (23.1%)	20 (76.9%)	0.96 (0.77-1.20)	0.684
Macroregion				
Lima y Callao	32 (20.8%)	122 (79.2%)	Ref.	Ref.
Center	4 (17.4%)	19 (82.6%)	1.04 (0.85-1.28)	0.743
North	5 (8.06%)	57 (91.9%)	1.16 (1.04-1.29)	0.021
East	15 (21.7%)	54 (78.3%)	0.99 (0.85-1.15)	0.863
South	20 (39.2%)	31 (60.8%)	0.77 (0.61-0.97)	0.012
Country of birth				
Perú	75 (21.2%)	278 (78.8%)	Ref.	Ref.
Other countries	1 (16.7%)	5 (83.3%)	1.06 (0.74-1.52)	0.864
Undergraduate studies in Peru				
No	4 (30.8%)	9 (69.2%)	Ref.	Ref.
Yes	72 (20.8%)	274 (79.2%)	1.14 (0.79-1.65)	0.404
Type of university where the undergraduate studies were carried out				
Private university	21 (17.2%)	101 (82.8%)	Ref.	Ref.
Public university	55 (23.2%)	182 (76.8%)	0.93 (0.83-1.03)	0.190
Years since university graduation				
<1	13 (31.7%)	28 (68.3%)	Ref.	Ref.
1-9	45 (18.7%)	196 (81.3%)	1.19 (0.96-1.48)	0.069
>9	18 (23.4%)	59 (76.6%)	1.12 (0.88-1.43)	0.339
Medical position held				
General Physician	47 (18.3%)	210 (81.7%)	Ref.	Ref.
Resident physician (in training)	12 (32.4%)	25 (67.6%)	0.83 (0.66-1.04)	0.058
Specialist physician	17 (26.2%)	48 (73.8%)	0.90 (0.77-1.06)	0.167
Master's degree or higher				
No	63 (20.9%)	238 (79.1%)	Ref.	Ref.
Yes	13 (22.4%)	45 (77.6%)	0.98 (0.84-1.14)	0.787
Sector in which they currently work				
Private	14 (15.9%)	74 (84.1%)	Ref.	Ref.
Public	54 (24.1%)	170 (75.9%)	0.90 (0.80-1.01)	0.114
Public and Private	8 (17.0%)	39 (83.0%)	0.99 (0.84-1.16)	0.860
Job location				
Rural	22 (26.2%)	62 (73.8%)	Ref.	Ref.

Urban	54 (19.6%)	221 (80.4%)	1.09 (0.95-1.25)	0.206
Have you heard about the increase of dengue cases in Peru?				
No	0 (0.00%)	1 (100%)	Ref.	Ref.
Yes	76 (21.2%)	282 (78.8%)	0.79 (0.75-0.83)	0.788
Have you read the ¨Peruvian clinical practice guide for the clinical management and treatment of Dengue" approved by Ministerial Resolution No. 071-2017-MINSA?				
No	48 (38.7%)	76 (61.3%)	Ref.	Ref.
Yes	28 (11.9%)	207 (88.1%)	1.44 (1.24-1.67)	<0.001
Have you received any training on the clinical management of dengue in Peru in the last three months?				
No	53 (27.9%)	137 (72.1%)	Ref.	Ref.
Yes	23 (13.6%)	146 (86.4%)	1.20 (1.08-1.33)	0.001
In your medical practice, have you ever treated patients with a confirmed or probable diagnosis of dengue fever?				
No	36 (45.0%)	44 (55.0%)	Ref.	Ref.
Yes	40 (14.3%)	239 (85.7%)	1.56 (1.27-1.91)	<0.001
Have you treated probable or suspected cases of dengue fever in the last 3 months?				
No	50 (37.0%)	85 (63.0%)	Ref.	Ref.
Yes	26 (11.6%)	198 (88.4%)	1.40 (1.22-1.61)	<0.001
How often have you treated cases of dengue fever?				
Not treated in the last 3 months	50 (37.0%)	85 (63.0%)	Ref.	Ref.
Rarely (1-10 cases/in the last three months)	9 (16.4%)	46 (83.6%)	1.33 (1.12-1.58)	0.004
Sometimes (1-10 cases/month)	12 (16.0%)	63 (84.0%)	1.33 (1.13-1.57)	0.001
Frequently (1- 10 cases/week or more)	5 (5.32%)	89 (94.7%)	1.50 (1.31-1.73)	<0.001
What is your opinion of the following statement? The Peruvian Ministry of Health is ADEQUATELY training physicians about dengue during the current outbreak.				
Neither agree nor disagree	28 (29.2%)	68 (70.8%)	Ref.	Ref.
Disagree	36 (20.9%)	136 (79.1%)	1.12 (0.96-1.30)	0.136
Agree	12 (13.2%)	79 (86.8%)	1.23 (1.05-1.43)	0.008
What is your opinion about the following statement? The MINSA is ADEQUATELY applying vector prevention and control measures (fumigation, elimination of mosquito breeding sites, etc.)				
Neither agree nor disagree	25 (25.0%)	75 (75.0%)	Ref.	Ref.
Disagree	37 (20.3%)	145 (79.7%)	1.06 (0.93-1.22)	0.370
Agree	14 (18.2%)	63 (81.8%)	1.09 (0.93-1.27)	0.286
What do you think of the following statement? The MINSA should implement vaccination against dengue in the country.				
Neither agree nor disagree	20 (29.4%)	48 (70.6%)	Ref.	Ref.
Disagree	11 (31.4%)	24 (68.6%)	0.97 (0.74-1.27)	0.830
Agree	45 (17.6%)	211 (82.4%)	1.17 (0.99-1.38)	0.037
What is your opinion of the following statement? The MINSA has the necessary resources to deal with dengue cases in the current outbreak.				
Neither agree nor disagree	19 (28.4%)	48 (71.6%)	Ref.	Ref.
Disagree	47 (20.7%)	180 (79.3%)	1.11 (0.94-1.30)	0.196
Agree	10 (15.4%)	55 (84.6%)	1.18 (0.98-1.42)	0.077

[1]Media (SD)

cPR: Crude Prevalence Ratio; CI: Confidence Interval; Ref: Reference. MINSA: Peruvian Ministry of Health.
